# Adrenal infarction secondary to methamphetamine use: a case report and review of the literature

**DOI:** 10.1186/s13256-022-03573-7

**Published:** 2022-10-18

**Authors:** Surendra Sapkota, Sarah David, Sandipa Sharma, Suraj Shrestha, Abhishek Kalla

**Affiliations:** 1 Department of Internal Medicine, Ascension Saint Agnes Hospital, 900 S Caton Ave, Baltimore, MD 21229 USA; 2 Ross University School of Medicine, Bridgetown, Barbados; 3 Shivanagar Primary Health Care Center, Chitwan, Nepal; 4 Maharajgunj Medical Campus, Institute of Medicine, Kathmandu, Nepal; 5 Ascension Saint Agnes Hospital, Cancer Institute, Baltimore, MD USA

**Keywords:** Acute abdomen, Adrenal infarction, Methamphetamine, Substance use

## Abstract

**Background:**

An acute abdomen can have a variety of causes. A commonly missed cause of abdominal pain is direct substance abuse and its sequelae. The use of methamphetamine is rising in the United States resulting in significant morbidity and mortality. There has been no reported case of methamphetamine-induced adrenal infarction based on an extensive review of available literature.

**Case presentation:**

We present a case of a 34-year-old Hispanic man who presented with acute abdominal pain secondary to adrenal infarction in the setting of methamphetamine use. Left paraumbilical tenderness was present on abdominal examination. Contrast-enhanced CT of the abdomen and pelvis revealed internal hypoenhancement of the left adrenal gland, consistent with acute left adrenal infarction. The patient was managed with enoxaparin and apixaban.

**Conclusion:**

Substance abuse, especially among young patients, can at times present with acute abdomen. This mandates physicians to be vigilant and take into consideration the history of substance abuse and relevant investigations. Timely diagnosis and management can prevent life-threatening complications.

## Background

Acute abdominal pain can have a variety of causes that include infectious, inflammatory, obstructive, anatomic abnormalities, or vascular causes along with many others. Some of the more common differentials include pancreatitis, appendicitis, trauma, and diverticulitis. A thorough history by the evaluating physician is necessary to narrow the differential diagnosis. A commonly missed cause of abdominal pain is direct substance abuse and sequelae of substance use [1]. Substances such as amphetamines, opioids, and cocaine can cause acute abdominal pain through a variety of mechanisms. Methamphetamine is a potent and highly addictive psychostimulant drug of abuse. According to the 2017 National Survey on Drug Use and Health, about 1.6 million people reported methamphetamine use [2]. Methamphetamine use is rising in the United States resulting in significant morbidity and mortality. Knowledge of methamphetamine-induced intestinal ischemia is limited to a few case reports. There are currently no reports of methamphetamine-induced adrenal infarction. We present a case of a 34-year Hispanic male with an adrenal infarction after methamphetamine use.

## Case presentation

A 34-year-old Hispanic male with a past medical history of hypertension presented to our hospital with abdominal pain that started the night before admission. The patient reported 10/10 intensity pain. He described his pain as constant, with no specific relieving factors, sharp, located mainly in the left paraumbilical region, and radiating to his left flank. He reported having nausea and three episodes of non-bloody, non-bilious emesis. He stated that his friends gave him crystal meth a day prior to the onset of his symptoms, adding that it was the first time in his life that he had ever taken illicit drugs. He denied fever, chills, cough, chest pain, or shortness of breath. He visited the clinic in our hospital about 5 years ago and was diagnosed with hypertension but was not taking any medications at the time of his emergency department visit. There was no history of any surgeries in the past. He had no history of any allergies to medications or foods. He reported that his father, mother, sister, and brothers were healthy, and all of them lived in Honduras. The patient's social history revealed that he worked as a painter and lived with his wife and kids. He denied smoking and prior illicit drug use. He reported occasionally drinking a few beers.

On admission, he had a temperature of 96.9 Fahrenheit, heart rate of 87 beats per minute, respiratory rate of 20/minute, and an oxygen saturation of 98% on room air. On physical examination, the patient appeared to be obese and in significant distress. There was left paraumbilical tenderness along with the guarding but no rebound tenderness. In the emergency room, the patient required multiple doses of intravenous morphine for pain relief.

**Fig. 1:**
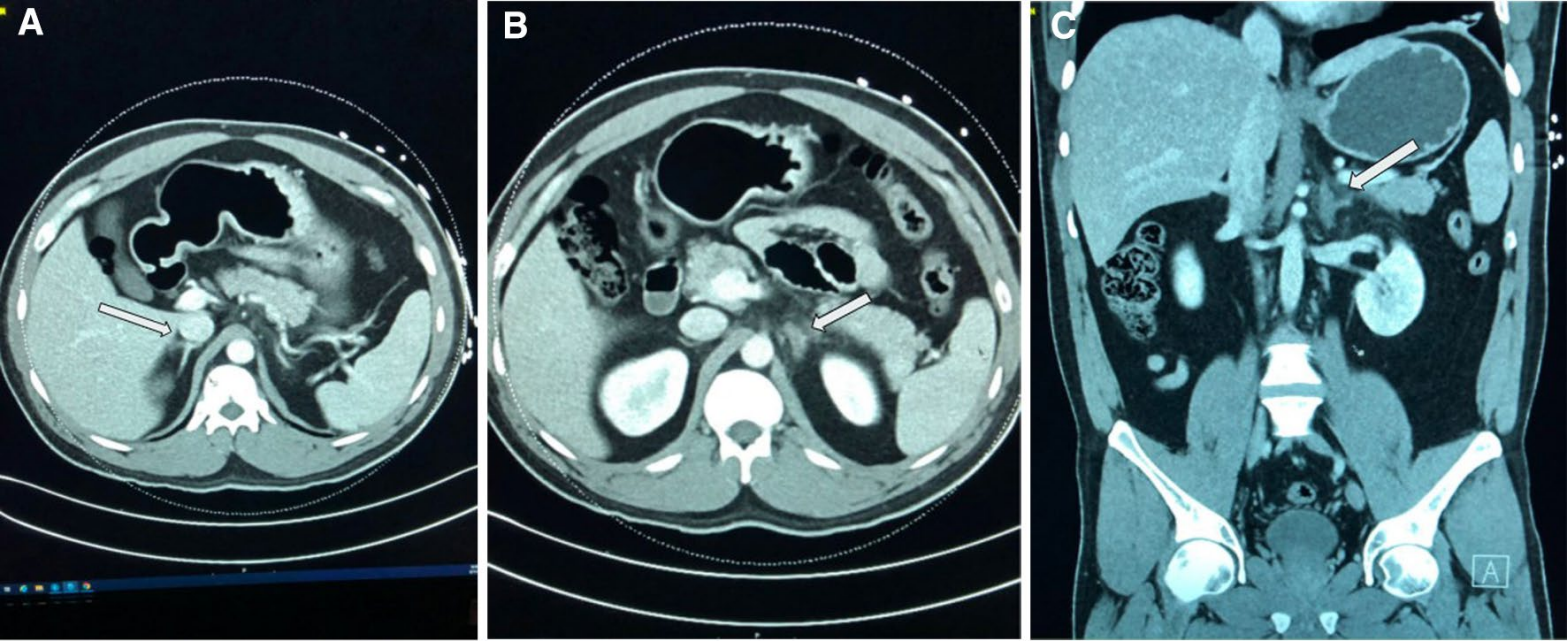
**A** Axial view of CT abdomen with arrow showing normal density and enhancement of the right adrenal gland. **B** Axial view of CT abdomen with arrow showing thickening of the left adrenal gland with inflammatory stranding of the surrounding fat, and internal hypoenhancement of the gland. **C** Coronal view of CT abdomen with arrow depicting findings as illustrated in **B**

Pertinent laboratory works on admission included: leukocytes 13,900/μL, hemoglobin 16.3 g/dL, platelet count 720,000/μL, creatinine 0.9 mg/dL, lipase 14 U/L, amylase 34 U/L. No significant red cell or platelet abnormalities were noted on the peripheral smear. (Table 1). Urine toxicology was positive for amphetamines and opiates. Urinalysis revealed 1 + ketones, 1 + protein, 3–5 red blood cells (RBCs), 0–2 white blood cells (WBCs) and was negative for bacteria, leukocyte esterase, and urine nitrite. The patient tested negative for COVID-19. Abdominal ultrasound showed no acute pathology. Computed tomography (CT) abdomen and pelvis with contrast showed thickening of the left adrenal gland with inflammatory stranding of the surrounding fat and internal hypoenhancement of the gland, consistent with acute left adrenal infarction (Fig. 1A–C). There was normal density and enhancement of the right adrenal gland.

**Table 1:** Laboratory parameters along with reference range at the time of admission

Labs	Values	Reference ranges
White blood cells	13.9 K/μL	4.0–11.0 K/μL
Red blood cells	5.7 M/μL	4.50–5.90 M/uL
Hemoglobin	16.3 g/dL	13.0–17.0 g/dL
Hematocrit	48.9%	41.0–50.0%
MCV	85.8 fL	80.0–99.0 fL
Platelet count	787 K/μL	150–400 K/uL
Venous blood gas (VBG) pH	7.34	7.31–7.41
VBG HCO_3_	28 meq/L	22–26 meq/L
VBG pCO_2_	53 mmHg	41–51 mmHg
Lactic acid	1.30 mmol/L	0.5–1.6 mmol/L
Sodium	137 mEq/L	136–145 mEq/L
Potassium	3.6 mEq/L	3.5–5.1 mEq/L
Blood urea nitrogen	13 mg/dL	8–21 mg/dL
Creatinine	0.9 mg/dL	0.6–1.2 mg/dL
Total bilirubin	0.6 mg/dL	< 1.5 mg/dL
AST	33 U/L	< 39 U/L
ALT	65 U/L	< 42 U/L
ALP	66 U/L	35–126 U/L
Lipase	14	23–300 U/L
Amylase	34	28–100 U/L

*AST* aspartate aminotransferase, *ALT* alanine aminotransferase, *ALP* alkaline phosphatase

The patient was admitted to the telemetry unit for close monitoring for possible hemorrhage or hypotension from the adrenal infarction. Hematology was consulted in the Emergency Department. Per hematology recommendations, the patient was started on a therapeutic dose of Lovenox (enoxaparin-1 mg/kg twice daily) and transitioned to eliquis (apixaban-5 mg twice daily) the next day. A transthoracic echocardiogram revealed a normal-sized cavity and normal wall thickness of the left ventricle with an ejection fraction of 60–65%, and normal diastolic function. The cavity size and systolic function of the right ventricle were normal as well. A bubble study was negative for an intracardiac shunt.

The patient’s pain was managed adequately with oxycodone and decreased significantly compared to the time of admission. He was discharged the next day in stable condition. In the setting of substance abuse, anticoagulation was considered for a minimum of 3 months (apixaban-5 mg twice daily), for the duration of treatment to be discussed on an outpatient basis. An outpatient appointment for hematology was arranged in a week after discharge.

## Discussion and conclusion

The adrenal gland has a unique vasculature that makes it prone to infarction in hypercoagulable states. The arterial supply is rich but venous drainage is limited to a single vein, and localization of thrombi in the adrenal vein might result in local stasis of blood leading to edema and necrosis of the gland [3]. Adrenal infarction is a very rare event but is occasionally seen in hypercoagulable states. Some of the common acquired factors for hypercoagulopathy are surgery or trauma, immobilization, underlying malignancies, estrogen replacement therapy, pregnancy, antiphospholipid antibody syndrome, heparin-induced thrombocytopenia, nephrotic syndrome, smoking, and inherited thrombophilia [4]. Common hereditary factors include factor V Leiden, prothrombin gene mutation, protein C and S deficiency, and antithrombin deficiency [4].

There have been reports of an increased incidence of adrenal infarction in pregnancy, due to physiologic changes in pregnancy such as increased blood volume, decreased production of anticoagulant factors, and venous stasis due to the mass effect of the gravid uterus. Pregnancy itself increases the risk of venous thromboembolism by approximately fivefold [5]. In a study by Glomski *et al*, unilateral non-hemorrhagic adrenal infarction was identified in 1.3% of abdominal magnetic resonance imaging (MRI) examinations performed for pregnant women with acute abdominal or flank pain [5].

The incidence of adrenal infarction in COVID 19 patients has also been described by Pierre *et al* in their retrospective cohort study. It was found that out of the 219 patients with critical (*n* = 52) and severe lung (*n* = 167) parenchyma lesions, 51 (23%) had CT scan signs of acute adrenal infarction, which was bilateral in 45 patients (88%). The presence of acute adrenal infarction in COVID-19 patients on initial chest CT was associated with a higher intensive care unit (ICU) admission rate and a longer hospital stay [6]. The mechanism is explained by increased activation of coagulation factors secondary to a systemic inflammatory response syndrome and/or hypovolemic states seen in patients with COVID-19 in septic shock [6].

Although limited, there is growing evidence of methamphetamine-induced thromboembolism. Methamphetamine is a potent stimulant with high abuse potential that can be smoked, snorted, injected, or taken orally [7]. Compared with other stimulants (e.g., cocaine and nicotine) the half-life of methamphetamine is relatively long, ranging from 8 to 12 hours with a rapid onset of activity [8]. Methamphetamine leads to increased levels of dopamine in the body which subsequently causes vasoconstriction by acting on alpha-1 adrenergic receptors in arteriolar smooth muscle. Additionally, it stimulates the release of endothelin-1, a potent vasoconstrictor that is one hundred times more potent than noradrenaline [8]. This vasoconstriction ultimately causes hypoxia and infarction of multiple organs like the heart and intestine. Multiple forms of cardiovascular disease have been reported with the use of methamphetamine. Javad *et al.* have described a unique presentation of methamphetamine-induced cardiomyopathy with multiple bilateral pulmonary emboli [9]. Rajesh *et al.* have described a case of methamphetamine cardiotoxicity presenting with acute pulmonary embolism and multiple, bi-ventricular thrombi [10]. A review of the Australian National Coronial Information System data showed that 6.5% of all non-accidental deaths in methamphetamine users are due to sudden cardiac death [8]. The use of methamphetamine has been associated with an increase in the rate of coronary artery disease, with myocardial infarction often observed in young patients with a history of methamphetamine abuse. Furthermore, there is growing evidence that methamphetamine can cause QT interval prolongation, which can induce cardiac dysrhythmias [8]. However, there has been no reported case of methamphetamine-induced adrenal infarction based on an extensive review of available literature.

Our patient also presented with an elevated platelet count, which could be an acute phase reactant. He had similarly elevated platelet counts when he presented to the emergency department with pneumonia 3 years ago. A Janus kinase 2 (JAK2) mutation panel was ordered. Urgent inpatient hypercoagulable work-up was not done due to the presence of acute infarction, active anticoagulation, and since the hypercoagulable results would not change the need for immediate anticoagulation. A hematology appointment was arranged at our center in a week of discharge; however, the patient didn’t follow up. JAK2 mutation, calreticulin mutation, and MPL mutation were all negative. In a telephone conversation a few weeks later after the discharge, the patient indicated that he was doing well. He was strongly encouraged to follow up with the primary care physician and the hematologist.

We described the case of a 34-year-old man who presented with acute abdominal pain secondary to adrenal infarction in the setting of methamphetamine use. This should alert physicians to consider questions about substance abuse as a part of history taking as prompt diagnosis and treatment of adrenal infarction with anticoagulation in the setting of substance abuse can prevent life-threatening complications and decrease associated mortality.

## Data Availability

All the required information is available in the manuscript.

## Abbreviations

CT: Computed tomography
ICU: Intensive care unit
JAK2: Janus kinase 2
MRI: Magnetic resonance imaging
RBC: Red blood cell
WBC: White blood cell
